# High Glucose Causes Distinct Expression Patterns of Primary Human Skin Cells by RNA Sequencing

**DOI:** 10.3389/fendo.2021.603645

**Published:** 2021-03-08

**Authors:** Shan Zhang, Zunxiang Ke, Chao Yang, Peng Zhou, Huanzong Jiang, Lei Chen, Yiqing Li, Qin Li

**Affiliations:** ^1^ Department of Vascular Surgery, Union Hospital, Tongji Medical College, Huazhong University of Science and Technology, Wuhan, China; ^2^ Institute of Hematology, Union Hospital, Tongji Medical College, Huazhong University of Science and Technology, Wuhan, China

**Keywords:** high glucose, RNA sequencing (RNA-Seq), human epidermal keratinocytes (HEKs), human dermal fibroblasts (HDFs), human dermal microvascular endothelial cells (HDMECs), diabetes

## Abstract

Diabetes-related skin problems represent the most common long-term complications in diabetes mellitus patients. These complications, which include diabetic dermopathy, diabetic blisters, necrobiosis lipoidica diabeticorum, and eruptive xanthomatosis, may dramatically impair patients’ quality of life and cause long-lasting disability. However, the cellular and molecular mechanisms linking diabetes-related hyperglycemia and skin complications are still incompletely understood. To assess the role of the various skin-cell types in hyperglycemia-induced skin disorders, we performed RNA sequencing-based transcriptome analysis, measuring gene expression patterns in biological replicates in normal- and high glucose-stimulated skin cells. Three primary human skin-cell types were examined, *i.e.*, epidermal keratinocytes, dermal fibroblasts, and dermal microvascular endothelial cells. For each separate cell type, we identified gene expression. Comparing gene abundances and expression levels revealed that transcription profiles exhibit distinct patterns in the three skin-cell types exposed to normal (*i.e.*, physiological) glucose treatment and high (*i.e.*, supraphysiological) glucose treatment. The obtained data indicate that high glucose induced differential gene expression and distinct activity patterns in signaling pathways in each skin-cell type. We are adding these data to the public database in the hope that they will facilitate future studies to develop novel targeted interventions for diabetic skin complications.

## Introduction

Diabetes mellitus (DM) is a highly prevalent metabolic disorder characterized by hyperglycemia and serious complications (1). Diabetes affects ∼9.5% of the world population, and its prevalence was estimated to increase by >50% by 2030 (2). The global increase in diabetes can be expected to pose medical, social, and economic burdens. At present, significant progress is being made in the treatment of diabetes. In addition, extensive attention is being paid to the management of organ complications caused by long-term hyperglycemia, including those in kidneys, eyes, heart, nerves, and skin. Diabetes-related dermal conditions include necrobiosis lipoidica, acanthosis nigricans, digital sclerosis, diabetic dermopathy, skin infections, and ulcers (3). Furthermore, delayed wound healing of diabetes is a major medical problem in the current treatment of diabetic patients and is receiving widespread attention in medical research. Although it is clear that certain skin lesions become more common and are among the first clinically observed signs of diabetes, the exact molecular mechanisms responsible for diabetic skin remain incompletely understood.

The skin is a complex organ, with a structure consisting of two main tissue layers: the overlying epidermis, which primarily contains keratinocytes, and the underlying dermis, a connective tissue that comprises collagens and fibers, along with multiple cell types such as fibroblasts, endothelial cells, macrophages, and mast cells (4, 5). Previous studies demonstrated that these skin cells are responsible for maintaining healthy skin tissue homeostasis and are critically involved in wound healing. Furthermore, more recent data indicate that dermal fibroblasts, microvascular endothelial cells, and epidermal keratinocytes may also influence the outcome of skin diseases in diabetes, in particular, diabetic wound healing (6). However, the exact molecular mechanisms whereby diabetes alters the physiological processes of the skin have not been elucidated. Developmentally, the epidermis originates from the ectoderm, while the dermis emerges from the mesoderm. Although closely related, epidermal keratinocytes, dermal fibroblasts and dermal microvascular endothelial cells have distinct developmental origins, fulfilling unique physiological roles. Thus, there is a need to illuminate how individual cell types function within skin tissue, and to clarify if induced changes in gene expression show similar or distinct patterns across the three cell types in diabetic skin.

Furthermore, patients with DM are also more likely to suffer from various cutaneous manifestations, including skin tightening, thickening, itch, infection, and impaired wound healing (7, 8). Although previous studies demonstrated that certain molecules are abnormally expressed in three cell types of skin under diabetic conditions, other studies indicated that some molecules were expressed differently and inconsistently by the three cell types. One study showed that exposure to high glucose (HG) was followed by increased expression of Notch intracellular domain in fibroblasts, microvascular endothelial cells, and keratinocytes (2). In addition, increased expression of MMP9 under hypoglycemic conditions has been detected not only in human skin fibroblasts and microvascular endothelial cells but also in keratinocytes (9, 10). However, comparisons of normal and diseased skin showed that some genes showed different patterns of differential expression in the three cell types. For instance, following 24 h of incubation with dexamethasone, estrogen receptor-beta was upregulated in cultured epidermal keratinocytes, whereas it was downregulated in dermal fibroblasts (11). Moreover, stromal cell-derived factor-1, which is mainly secreted by dermal fibroblasts and acts as a chemokine, functions as a mitogen in the epidermis, particularly in keratinocyte proliferation (12). Observations such as these suggest that the effects of hyperglycemia on skin fibroblasts, microvascular endothelial cells, and keratinocytes may differ too. This raises the question to which extent shifts in gene expression under HG-induced conditions in these three cell types overlap.

To address this issue, we used RNA sequencing (RNA-Seq) technology to investigate genome-wide changes in gene expression in major skin-cell types during stimulation with HG and normal glucose (NG). For three of these cell types, *i.e.*, dermal fibroblasts, microvascular endothelial cells, and epidermal keratinocytes, we identified several genes that are expressed at physiological glucose levels. Furthermore, we generated comprehensive datasets of genes and pathways whose transcriptional profiles change under HG conditions. Overall, this study was intended to provide an abundant resource that may help improve the understanding of molecular and cellular mechanisms underlying diabetes-related dermatological problems. It may furthermore guide future research of molecular targets for novel therapeutic intervention of diabetic skin complications.

## Materials and Methods

### Cell Culture and Sample Preparation

Primary HDF (Cat # 2320), HDMEC (Cat # 2020), and HEK (Cat # 2110) were obtained from ScienCell and cultured in their special medium provided by ScienCell. Primary HDFs were cultured in a fibroblast medium (8 mM glucose), supplemented with 2% fetal bovine serum (FBS) and 1% fibroblast growth supplement. Primary HDMECs were cultured in an endothelial cell medium (5.55 mM glucose), supplemented with 5% FBS and 1% endothelial cell growth supplement. Primary HEKs were cultured in a fibroblast medium (6 mM glucose) supplemented with 1% keratinocyte growth supplement. All media were supplemented with 100 IU/ml penicillin. All types of the cells were maintained at 37°C in a 5% CO_2_ humidified incubator, and cells from the fourth to ninth passages were used for experiments. In the experiments, cells were initially plated on 3-cm plates in their special medium. We uniformly define the glucose concentration of their special medium as normal glucose (NG). At 80% confluence, the cells were switched to HG (30 Mm) medium for 24 h. For each condition, three biological replicates were generated.

### Total RNA Extraction and RNA Sequencing Procedures

RNAs from cultivated skin cells were extracted using RNAiso Plus (Cat # 9108, TaKaRa). Extracted RNA was measured and checked for concentration, purity, and integrity of RNA on a Fragment Analyzer Bioanalyzer using a standard sensitivity RNA analysis Kit. RNA-Seq was then performed on a commercially available service (service ID # F19FTSCCWLJ6421, BGI, Wuhan, China). Briefly, after poly (A)-based mRNA enrichment with oligo (dT) magnetic beads using 18 total RNA samples as the input material, the mRNA was fragmented. After the first and second strands, cDNA synthesis, end-repair, 3′-adenylation, and ligation of the fork-tailed adapters were performed. Following PCR amplification, library-quality was verified on a Fragment Analyzer bioanalyzer for a fragment size of 150 bp and for concentration. Paired-end libraries were sequenced using the BGISEQ-2000 (2 × 150 nucleotide read length) with a mean sequencing coverage depth of up to 300 ×, and coverage of the target region close to 99.9%. Meanwhile, the FASTQ data has been deposited in the CNGB Nucleotide Sequence Archive (CNSA) repository (CNP0000999).

### Quality Control and Alignment of Sequencing Datasets

Sequencing of cDNA libraries generated over 41 to 53 million paired reads per sample. Low-quality reads of FASTQ files were filtered using SOAPnuke software v1.5.2 to obtain clean reads. After filtering, the high-quality reads were aligned against the human reference genome (GRCh38.p12) using an RNA-Seq-spliced read mapper HISAT2 (version 2.0.4). FPKM values for gene expression levels were calculated for annotated RefSeq genes using RSEM (version 1.2.8). To estimate the heterogeneity of our samples, we performed Pearson correlation coefficient analysis and PCA, both using R software, to map clusters within the gene expression profile from all 18 samples. Normalization and differential expression analyses were estimated from count data using DESeq2. The heat map analysis was performed with the “pheatmap” R package with default parameters selecting the DEGs after FDR-corrected *p* values (*q* values ≤ 0.05) and fold changes ≥1.2.

### Functional Enrichment Analysis

GO and KEGG enrichment analysis of DEGs was performed using the Dr. TOM approach, which is an online database of BGI. The GO and KEGG functional enrichment apply the hypergeometric test to find GO term and Pathway that are significantly enriched in candidate genes’ map to the entire genome background. Subsequently, *q* value was conducted. We performed bubble diagram to show the significantly enriched KEGG signaling pathway and GO. X-axis represents the Rich Ratio, which means the ratio of selected gene number annotated to a particular item to the total gene number annotated in this item. The calculating formula is Rich Ratio = Term Candidate Gene Num/Term Gene Num. Y-axis represents GO term or KEGG Pathway. The GO terms and KEGG pathways of which *q* value ≤ 0.05 were defined as significantly enriched.

### Protein–Protein Interaction Network Construction and Analysis

To estimate the interactive relationship of DEGs, we constructed PPI networks using the online tool STRING (13, 14).

### Statistics

Results are presented as fold change. GO terms or KEGG pathways with *q values* (corrected *p values*) ≤0.05 were considered to be significantly enriched by candidate genes. All the figures were performed using GraphPad Prism 7.0 and Adobe Illustrator.

## Results

### Exprimental Design of RNA Sequencing

Human dermal fibroblasts (HDFs), human dermal microvascular endothelial cells (HDMECs), and human epidermal keratinocytes (HEKs) are the main contributors to diabetic dermopathy; therefore, we used these three cell types as models to assess the impact of hyperglycemia on the condition of the skin. To investigate transcriptome abundance, RNA-Seq was performed in primary HDF, HDMEC, and HEK exposed to either NG or HG (30 mM) medium for a duration of 24 h (**Supplemental Figure 1A**). To ensure the quality of the RNA obtained for sequencing, we evaluated its purity, concentration, and integrity (**Supplemental Table 1**). Next, RNA library construction, sequencing, and enrichment analysis were performed (**Supplemental Figure 1B**). The final dataset contained 41 to 53 million raw reads per sample. Following the filtering of the raw reads, sequencing showed that the average sample RNA-Seq library resulted in 42 million clean reads with an average mapping rate of 92% when aligned to human genomes (**Supplemental Table 2**).

### Feature Analysis of the Full Sequencing Data

For each cell type, the biological duplicates yielded reproducible normalized gene expression results showing Pearson correlation coefficients (R) in the following range: 0.90<R<0.99 (**Supplemental Figure 2A**), indicating excellent experimental specificity and technical reproducibility. To explore the ontogeny and relationships among the three cell types, we performed dimension reduction and clustering using Principal Component Analysis (PCA) (**Supplemental Figure 2B**). Reassuringly, the biological replicates from each cell type were found to cluster. Next, we used the Fragments per Kilobase of exon per Million mapped reads (FPKM) method to normalize the dataset of RNA-Seq expression levels. To identify the distribution of gene expression levels in each sample, we created Boxplots showing the degree of dispersion of the data distribution (**Supplemental Figure 2C**). To better delineate patterns of gene abundance, we created density maps of FPKM, thereby visualizing more clearly the range of gene expression levels (**Supplemental Figure 2D**). To visualize the number of genes within the different FPKM ranges, we examined three ranges, namely FPKM ≤1, FPKM 1 ~10, and FPKM ≥10 (**Supplemental Figure 2E**). As evident from the figure, the numbers in the FPKM ≤1 range account for a large proportion of gene expression variability. Overall, these outcomes confirmed the reliability of our sequencing data.

### Bioinformatic Analysis of Ubiquitously Expressed Genes in the Three Skin-Cell Types

**Figure 1:**
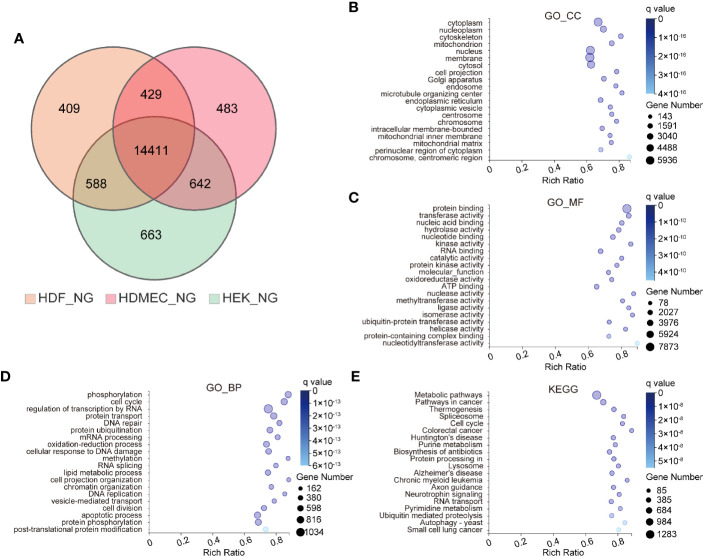
Bioinformatics analysis of ubiquitously expressed genes among three kinds of human skin cells. **(A)** Venn diagrams depicting the numbers of genes identified in HDF, HDMEC, and HEK under normal glucose stimulation. **(B–D)** Genes identified in all type cells are classified as ubiquitously expressed, whereas those identified in single-cell types were classified as cell type-specific. GO term analyses were performed for ubiquitously expressed genes. And GO analysis containing three sub-ontologies. CC for cellular component **(B)**, MF for molecular function **(C)**, and BP for biological process **(D)**. The color and size of the bubble represent the enriched q value and the number of genes, respectively. **(E)** Bubble chart of top 20 KEGG enrichments involving ubiquitously expressed genes.

To investigate the differences and similarities among HDF, HDMEC, and HEK, we performed transcriptomic profiling of these cell types under physiological conditions. Genes were classified as ubiquitously expressed or specifically expressed *via* entropy filtering. We found that a larger proportion of the identified genes were globally rather than specifically expressed, based on a total of 14,411 identified genes that were expressed in all cell types taken together (**Figure 1A**). To gain insight into the biological roles of the globally expressed genes, gene ontology (GO)-term and the Kyoto Encyclopedia of Genes and Genomes (KEGG) pathway analyses were conducted. At the level of the GO cellular component (GO_CC), the top three enriched items included cytoplasm, nucleoplasm, and cytoskeleton (**Figure 1B**), whereas protein binding, transferase activity, and nucleic acid binding were most enriched in the GO molecular functions component (GO_MF) (**Figure 1C**). At the level of GO biological process (GO_BP), phosphorylation, cell cycle, and regulation of transcription by RNA polymerase II were highly enriched (**Figure 1D**). KEGG analysis suggested that the most significantly altered pathways comprised metabolic pathways, pathways in cancer, and thermogenesis (**Figure 1E**).

### Bioinformatic Analysis of Cell Type-Specific Expression Genes

**Figure 2:**
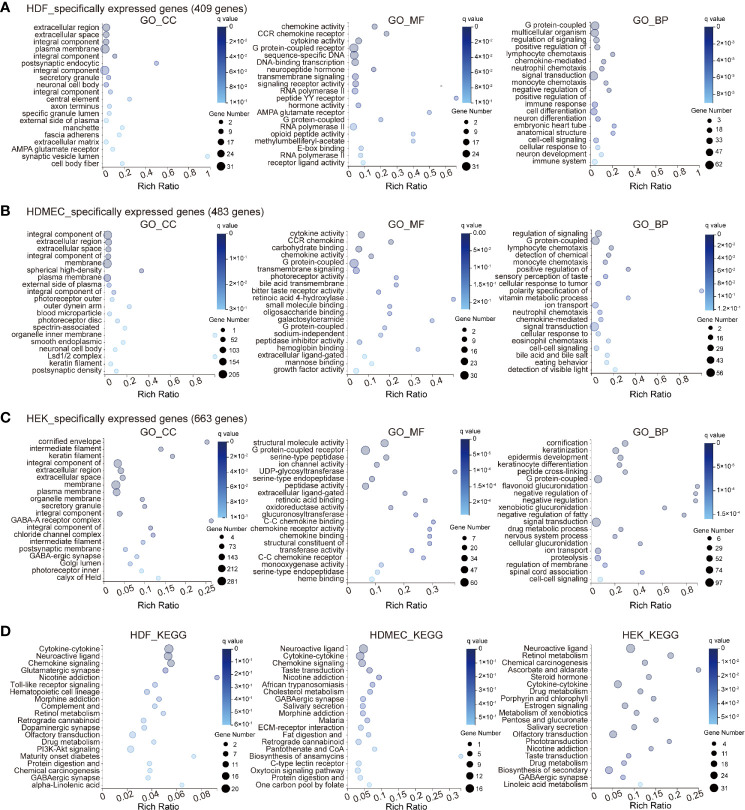
Bioinformatics analysis of cell type specificity of HDF, HDMEC, and HEK genes. **(A–C)** GO term analysis of genes specifically expressed in HDF **(A)**, HDMEC, **(B)** and HEK **(C)** cells. Enriched GO terms for cellular component, molecular function, and biological process are shown. The color and size of the bubble represent the enriched q value and the genes number, respectively. **(D)** Bubble chart of top twenty KEGG enrichments involving specifically expressed genes. Left, HDF; middle, HDMEC; right, HEK.

In addition to the analysis of the genes globally expressed across the three cell types, we included genes that were specifically expressed, reflecting heterogeneity among the three cell types. In the GO_CC category, HDF specifically expressed several genes involved in the extracellular region, extracellular space, and integral components of the plasma membrane. In the GO_MF category, HDF cells highly expressed genes involved in chemokine activity, CCR chemokine receptor binding, and cytokine activity, whereas in the GO_BP category, genes involved in G protein-coupled receptors, multicellular organism development, and regulation of signaling receptor activity were represented strongly (**Figure 2A**). Cell-specific gene representation in HDMEC was significantly enriched in cell type-specific genes related to integral components of the cell membrane, extracellular region, and extracellular space for the GO_CC category, whereas, cytokine activity, CCR chemokine receptor binding, and carbohydrate binding were mainly enriched for the GO_MF category. Within GO_BP, highly enriched terms included regulation of signaling receptors, G protein-coupled receptor, and lymphocyte chemotaxis (**Figure 2B**). It is worth noting that genes specifically expressed in HEK were significantly enriched in the cornified envelope, intermediate filament, and keratin filament in the GO_CC category. Meanwhile, GO_BP-related genes in HEK were specifically enriched in structural molecule activity and G protein-coupled receptor activity; moreover, serine-type peptidase activity was most enriched among molecular functions and for the GO_BP category, the most enriched terms were cornification, keratinization, and epidermis development (**Figure 2C**). We further compared the KEGG pathway of specifically expressed genes among the different cell types. The results showed that the cell type-specific expression pattern of HDF was significantly enriched in cytokine–cytokine receptor interaction, neuroactive ligand–receptor interaction, and chemokine signaling pathway, which is similar to HDMEC. In contrast, the cell type specificity of HEK was significantly enriched in neuroactive ligand-receptor interaction, retinol metabolism, and chemical carcinogenesis (**Figure 2D**).

### Identification of Overlapping, Differentially Expressed Genes in Human Dermal Fibroblast, Human Dermal Microvascular Endothelial Cells, and Human Epidermal Keratinocytes

**Figure 3:**
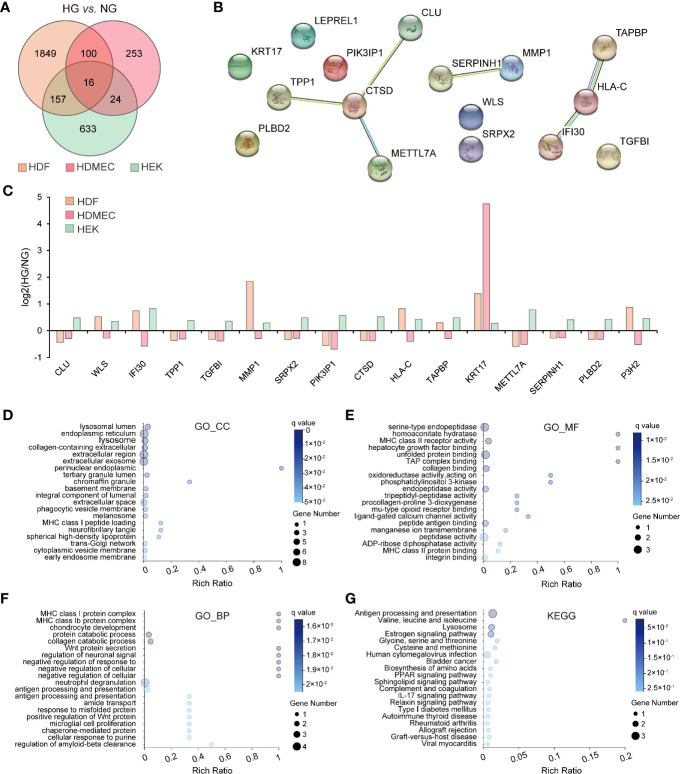
Bioinformatics analysis of overlapping DEGs among three cell types. **(A)** Venn diagram mapping intergroup comparisons (HDF_NG *vs*. HDF_HG, HDMEC_NG *vs*. HDMEC_HG, HEK_NG *vs*. HEK_HG) showing the number of differentially expressed genes with a *q value ≤*0.05 and fold change ≥1.2) **(B)** Protein interaction network of 16 overlapping genes showing mutual interactions. Each node indicates the individual gene, and edges indicate the protein–protein interactions among the overlapping genes. **(C)** Histogram depicting the logbase two-fold change for overlapping genes in the three cell types, showing up- and downregulation in separate columns. The orange columns represent HDF; red columns represent HDMEC; green columns represent HEK. **(D–F)** GO enrichment analysis of overlapping genes in the three cell types. Enriched GO terms for cellular component **(D)**, molecular function **(E)**, and biological process **(F)** are shown. **(G)** KEGG analysis of overlapping genes across the three cell types.

Given the different gene expression patterns in the three skin-cell types under physiological conditions, we wondered whether HG-induced shifts in gene expression are also similar in these cell types. Therefore, we compared the effects of HG stimulation on DEGs across cell types. When subjected to filtering with statistical tests (*q value* ≤ 0.05) and fold change thresholds (≥1.2), the HDF was found to have a total of 2,122 DEGs, whereas the HDMEC and HEK cells yielded 393 and 860 of such genes, respectively. Overall, the majority of these DEGs were observed exclusively in single-cell types, while only 16 genes were found equally in all cell types (**Figure 3A**). To elucidate the biological processes regulated by HG levels, we performed a network analysis on the 16 overlapping genes whose expression was changed in HDF, HDMEC, and HEK. We gathered protein–protein interaction data among the 16 genes by means of the Search Tool for the Retrieval of Interacting Genes/Proteins (STRING) database (15). We determined densely connected genes underlying protein–protein interactions within the network to identify nodes predicted to be involved in specific regulatory processes using functional enrichment analysis. This identified cathepsin D (CTSD) protein as the most highly connected nodes among 16 overlapping genes in three cell types (**Figure 3B**). Subsequently, we provided histograms of the overlapping genes expression, which were up- and downregulated in response to the HG treatment, separately displayed per cell line and direction of change (**Figure 3C**). Remarkably, Keratin 17 (KRT17) is the only gene whose expression was upregulated consistently in all cell types, while the others were regulated in diverse directions. To understand better the functions of overlapping genes, we performed GO enrichment analysis and KEGG pathway classifications. For the GO_CC category, the top three enriched items were lysosomal lumen, endoplasmic reticulum, and lysosome (**Figure 3D**), whereas serine-type endopeptidase activity, homoaconitate hydratase activity, and MHC class II receptor activity were the most enriched in the GO_MF category (**Figure 3E**). We furthermore observed high values for GO_BP, such as MHC class I and class Ib protein complex assembly and chondrocyte development (**Figure 3F**). Moreover, these genes were mainly enriched in antigen processing and presentation and lysosome signaling pathway (**Figure 3G**).

A strong relationship between DEGs and cell type-specific expression genes has been reported in previously published studies (16, 17). This urges us to study the cell type-specific gene expression changes in our sequencing data. However, the results suggested that a handful of DEGs was cell type-specific in each skin-cell type following HG stimulation. And the proportion of overlapping genes in cell type-specific expression genes was 3.91% in HDF, 0.83% in HDMEC, and 2.56% in HEK respectively (**Supplemental Figure 3**). Thus, the subsequent bioinformatic analyses of HG-induced DEGs in each cell type were performed to elucidate the potential mechanisms underlying diabetic skin complications.

### Identification of High Glucose-Induced Changes in Human Dermal Fibroblast

**Figure 4:**
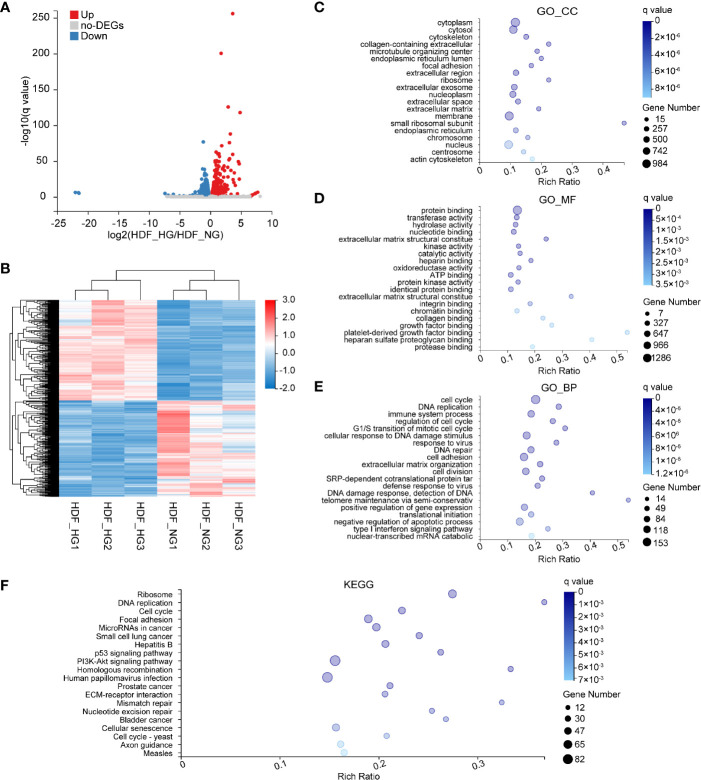
Bioinformatics analysis of DEGs in HG-induced HDF. **(A)** Volcano plots representing up- and downregulated DEGs of HDF in response to high glucose stimulation conditions. The size of each data point has been plotted against the negative log_10_ of the *q value* × log_2_ of the fold change, *q value ≤*0.05, and fold change ≥1.2. Red dots represent upregulated genes; blue dots represent downregulated genes. **(B)** Heat map of normalized expression values across all samples for genes differentially expressed between HDF_HG (n = 3) and HDF_NG (n = 3). Red and blue represent high and low expression, respectively. **(C–E)** GO enrichment analysis of differentially expressed genes in normal and high glucose-induced HDF. Enriched GO terms are shown for cellular component **(C)**, molecular function **(D)** and biological process **(E)**. **(F)** KEGG enrichment of differentially expressed genes in HDF.

To gain comprehensive, more precise insights into the effects of HG levels on HDF, we analyzed the transcription profiles of dermal fibroblasts in NG and HG conditions and screened for DEGs. Of the resulting genes, we selected those whose expression increased or decreased by more than 1.2-fold in HG-induced HDF, with a *q* value of ≤0.05; this yielded 2,122 DEGs (**Figure 4A**), 1,077 of which were upregulated and 1,045 downregulated. Next, a hierarchical cluster analysis of biological replicates was performed to visualize the experimental reproducibility of the selected genes (**Figure 4B**). Genes are color-coded according to their normalized read counts, which is a relative measure for gene expression, and the highlighted red areas and blue areas represent the high and low expression, respectively. Next, the identified genes were subjected to GO-annotation and KEGG pathway analysis. Cytoplasm, cytosol, and cytoskeleton were the most enriched terms in the GO_CC category (**Figure 4C**). The most highly represented terms of the GO_MF category were protein binding, transferase activity, and hydrolase activity (**Figure 4D**). In the GO_BP category, the cell cycle, DNA replication, and immune system process were the most highly enriched items (**Figure 4E**). KEGG pathways, including ribosome, DNA replication, and cell cycle signaling pathways, were among the most enriched (**Figure 4F**).

### Identification of High Glucose-Induced Changes in Human Dermal Microvascular Endothelial Cells

**Figure 5:**
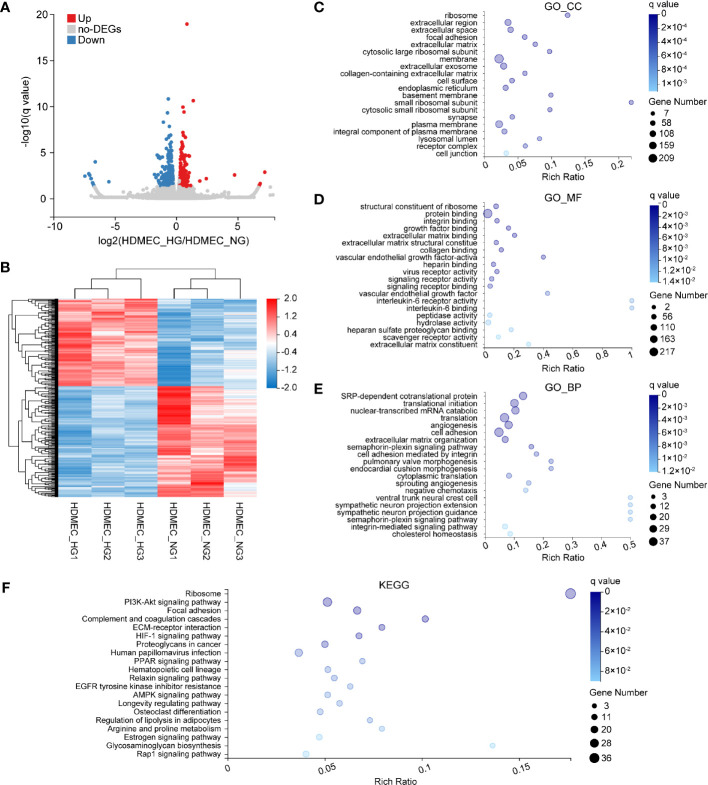
Bioinformatics analysis of DEGs in HDMEC following HG stimulation. **(A)** Venn plots depicting upregulated (red) and downregulated (blue) DEGs of HDMEC across two glucose-stimulation conditions. **(B)** Heat map of 393 genes that were differentially expressed between HDMEC_HG (n = 3) and HDMEC_NG (n = 3). Red and blue represent high and low expression, respectively. **(C–E)** GO enrichment analysis of differentially expressed genes in normal and high glucose-induced HDMEC. Enriched GO terms are shown for cellular component **(C)**, molecular function **(D)**, and biological process **(E)**. **(F)** KEGG enrichment of differentially expressed genes in HDMEC.

Next, we performed analyses on HDMEC, which exhibited the smallest number of DEGs. A total of 393 genes were filtered to be differentially expressed between HDMEC_NG and HDMEC_HG, among which 173 were upregulated and 220 were downregulated (**Figure 5A**). Furthermore, hierarchical cluster analysis was performed based on normalized read counts reflecting gene expression, following log z-transformation in the row direction (**Figure 5B**). To identify the biological functions in which these DEGs partake, we performed GO enrichment analysis. In the GO_CC category, the most abundant genes were associated with ribosome, extracellular region, and extracellular space (**Figure 5C**). Within the GO_MF category, the genes were mostly associated with the structural constituent of ribosome, protein binding, and integrin binding (**Figure 5D**). Moreover, SRP-dependent co-translational protein targeting the membrane, translational initiation, and nuclear-transcribed mRNA catabolic process were the main groups within the GO_BP category (**Figure 5E**). A KEGG pathway enrichment analysis was also carried out, and its results showed that most of the DEGs were enriched in the ribosome, PI3K–Akt signaling, and focal adhesion signaling pathways (**Figure 5F**).

### Identification of High Glucose-Induced Changes in Human Epidermal Keratinocytes

**Figure 6:**
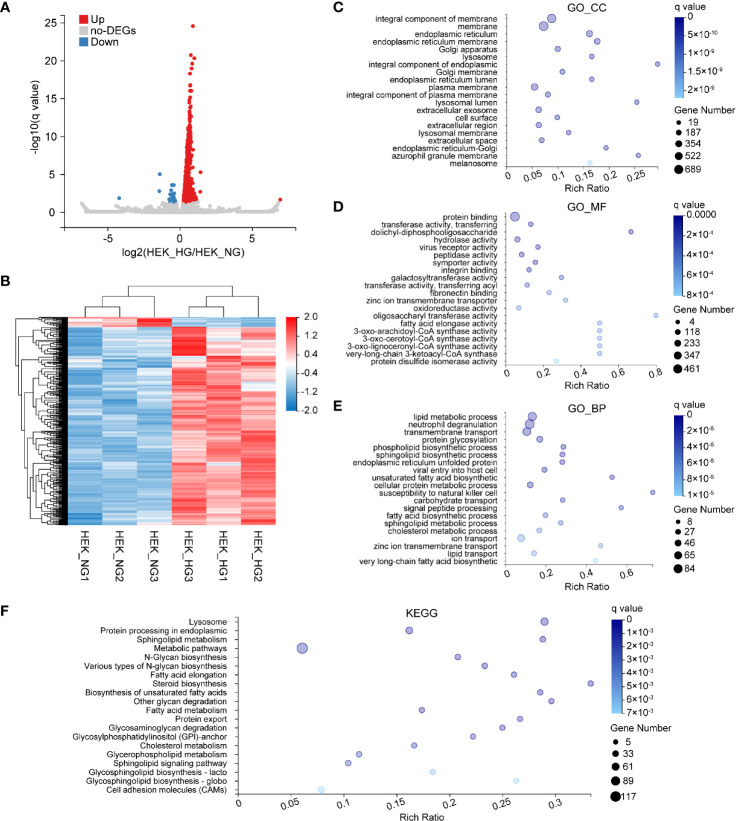
Bioinformatics analysis of DEGs affected by HG in HEK. **(A)** Volcano plots representing significant DEGs (*q value ≤*0.05, fold change ≥1.2) in HEK_HG *versus* HEK_HG. Red dots represent upregulated genes; blue dots the downregulated ones. **(B)** Heat map of 860 genes differentially expressed between HEK_HG (n = 3) and HEK_NG (n = 3). Red represents high expression, and blue represents low expression. **(C–E)** GO enrichment analysis of differentially expressed genes in normal and high glucose-induced HEK. Enriched GO terms are shown for cellular component **(C)**, molecular function **(D)** and biological process **(E)**. **(F)** KEGG enrichment of differentially expressed genes in HEK.

Subsequently, we examined DEGs involved in HEK cells. We finally identified 860 DEGs, 820 of which were upregulated, and 40 were downregulated (**Figure 6A**). To visualize the experimental reproducibility, we performed hierarchical clustering (**Figure 6B**). To evaluate their biological roles, GO enrichment analysis and KEGG pathway classifications were performed. The GO_CC category was most enriched for the integral component of the membrane, the membrane, and the endoplasmic reticulum (**Figure 6C**). For the GO_MF, the protein binding, transferase activity, and glycotransferase activity were highly represented (**Figure 6D**). Additionally, these DEGs were similarly enriched in GO_BP categories, such as the lipid metabolic process, neutrophil degranulation, and transmembrane transport (**Figure 6E**). The KEGG pathway enrichment analysis demonstrated that most of the genes were enriched in the lysosome, protein processing, and sphingolipid metabolism (**Figure 6F**).

## Discussion

In this study, we generated abundant transcriptome datasets that can be interrogated for a detailed understanding of the molecular and cellular bases of differences between normal and diabetic skin. We compared the gene expression profiles of separated cohorts of HDF, HDMEC, and HEK, which revealed distinct differential gene expression patterns. Specifically, for physiological conditions, we found that a larger proportion of the identified genes were expressed uniformly instead of specifically by three cell types. In addition, we globally compared transcriptome profiles in skin cells upon stimulation with two different levels of glucose. We observed many DEGs in the different cell populations. Importantly, it is plausible that certain genes and pathways are differentially regulated across cell types because of their distinct roles in dermal homeostasis. Moreover, these data may provide further insights into the transcriptional organization of the skin cells as well as skin biology and -development. In addition, predictive biomarker panels could be identified that could improve tissue analyses of normal and diabetic skins.

When transcriptome profiles were compared across the different skin-cell types under physiological conditions, no significant differences were found. KEGG analysis revealed global patterns of gene expression associated with metabolism and thermogenesis, which presumably enable the skin to perform normal physiological functions. Clearly, genes specifically expressed in a certain cell type are likely to be closely related to its unique physiological functions. Importantly, in the examination of the list of KEGG pathways, we noted that fibroblasts and endothelial cells were associated with immune system functioning (namely, cytokine–cytokine receptor interaction and chemokine signaling pathway). Consistent with previously published studies, the abnormal expression of cytokines, such as IL-20 and IL24, was also found in the skin of patients with dermopathy (18, 19). In contrast, the clearest trend in keratinocytes was a significant enrichment of specific genes in pathways related to metabolism. Our observations in the skin cells correspond to the anatomical and physiological diversity of the skin cells across dermal and epidermal tissue layers. Moreover, while the membrane and extracellular region were the major cellular components of fibroblasts and endothelial cells, the cornified envelope and intermediate filament were found to be a critical cellular component for keratinocytes, consistent with previous reports (20).

We then compared cells stimulated by two glucose levels (HG *vs*. NG) and observed a distinct transcriptional variation within all cell types. Unexpectedly, the three cell types had only 16 DEGs in common, most prominently in the lysosome signaling pathway. Interestingly, KRT17 is the only gene that was upregulated consistently in all three examined cell types, while the other identified genes were regulated in diverse directions in the different cell types. The lysosome was initially considered the cell’s “suicide bag” that degrades and processes cellular waste (21). Lysosomes are now recognized as signaling hubs that are crucially involved in energy metabolism and are key regulators of cell homeostasis (22, 23). However, the potential impact of lysosomal signaling in diabetic skin requires further verification. Surprisingly, the proportion of overlapping genes to DEGs was low, *i.e.*, 0.75%. In view of this observation, we analyzed the DEGs of all cell types separately. A clear pattern observed in fibroblasts was the significant enrichment of DEGs in the cell cycle and DNA replication signaling pathways. During the proliferative phases of wound healing, fibroblasts migrate into the wound site and differentiate into myofibroblasts, which synthesize and remodel the extracellular matrix to promote wound contraction (24). The observed effects of hyperglycemia on the cell cycle and DNA repair may be one of the mechanisms responsible for delayed wound healing in diabetes. The clearest trend found in fibroblasts was the significant enrichment of DEGs in the inflammation and infectious disease pathways. The family of 2′, 5′ oligoadenylate synthetases (OAS; 2′, 5′ AS), including OAS1, OAS2, OAS3, and OASL, are generally upregulated in HDF (**Supplemental Figure 4**). Comparing with previous study, the expression levels of OAS3 and OASL were also significantly increased in whole blood of diabetic patients (25). Meanwhile, this finding is in line with results obtained by Wu et al. (26), and our results further indicate that this upregulation mainly occurs in the fibroblasts. Moreover, consistent with data from the literature (10), we detected an elevated MMP9 expression in HDF after stimulation by HG levels (**Supplemental Figure 5**). Previous studies have observed increased MMP9 in chronic wounds (10, 27), which is thought to produce prolonged inflammation in diabetic wound healing models. The clearest trend in microvascular endothelial cells was the significant enrichment of DEGs in the immune and endocrine system pathways, such as the phosphatidylinositol 3-hydroxy kinase/protein kinase B (PI3K/AKT) signaling pathway. PI3K/AKT signaling is significant in the normal wound healing process and plays a vital role in the proliferation, regeneration, remodeling, and reepithelialization of injured tissue (28–30). In addition, much evidence points to a critical role of the PI3K/AKT pathway in wound healing in diverse pathological states, including hyperglycemia (31, 32). We found that the DEGs in HEK were significantly enriched in the metabolic pathway, especially lipid metabolism and glycan metabolism, and that the formyl peptide receptor 1 (FPR1) was significantly upregulated (**Supplemental Figure 6**). FPR1 is a member of the family of G protein-coupled receptors and plays a role following tissue damage (33). The FPR1 signal induces the production of reactive oxygen species (34), which is also a mediator of necroptosis (35). In a more recent study, Saito et al. reported that necroptosis in keratinocytes can be triggered *via* the RIP1/RIP3 complex by the interaction of Annexin A1 and FPR1 (36). Another study indicates that the level of FPR1 in diabetic mice is elevated and directly impaired glucose homeostasis (37). Annexin A1/FPR1 are candidate markers of disease occurrence and may be promising therapeutic targets.

Our study identified KRT17 as the only gene whose expression was upregulated in all three skin-cell types. KRT17 is a type I intermediate filament, which is expressed in the basal cells of epithelia. As a multifaceted cytoskeletal protein, KRT17 regulates multiple biological processes, including cell proliferation, migration, differentiation, inflammatory, and immune responses. Abnormal expression of KRT17 has been found in various dermatoses, including psoriasis, alopecia, and pachyonychia congenita (PC) (38). The genetic mutation in KRT17 is related to PC, which mainly manifests itself as hyperkeratosis of the nails, palm, and pelma (39). Meanwhile, microarray analysis of non-healing chronic ulcers identified that the KRT17 gene was significantly downregulated relative to expression levels in healing chronic ulcers (40), suggesting that dysregulation of KRT17 is involved in defective healing. Another research found KRT17 was significantly enriched in diabetic foot ulcers when compared to oral mucosa (41). In addition, keratin supplementation has been shown to speed wound healing and epithelialization in mice (42, 43). In general, abnormal expression of KRT17 was found to play a key role in a variety of skin diseases. This time, we first identified the overexpression of KRT17 in diabetic skin. In conclusion, KRT17 holds great potential as a future target for the treatment of diabetic dermopathy.

Here, we conducted transcriptomic profiling on various skin-cell types and found diverse patterns of gene expression both in NG and HG stimulation. However, the generalizability of these results is subject to certain limitations. Firstly, our experiments selected the major cellular components of the skin, including keratinocytes, fibroblasts and endothelial cells. However, it has been reported that besides these cells, melanocytes also play a prominent role in dermopathy (44, 45). Secondly, the experiments in this study were only performed in cell-based *in vitro* models. The differences between *in vitro* and *in vivo* need to be considered, further work needs to be done to establish whether the significant genes and pathways of our findings were involved in real-life conditions. Thirdly, the three cell types were cultured in different base media, which may cause certain influence on the results. Therefore, these findings need to be interpreted with caution in skin problems associated with diabetes mellitus. Moreover, even if 16 overlapping genes were differentially expressed in all the three examined skin-cell types, most of them exhibited inconsistent directions of gene expression changes, which is far beyond our expectations. In addition, previous studies have often focused on entire tissues, containing complex mixtures of cells. Whether such approaches provide accurate information about transcription patterns in different cells is debatable. Because of technological advances, more and more scholars have reverted to single-cell sequencing, through which, researchers have become aware of the heterogeneity of the cells and started to pay attention to the behavior of single cells and cell types rather than entire tissues. Some challenges remain in single-cell RNA-Seq, in which measurements are highly sensitive to the efficiency of required enzymatic steps. Additionally, the required amplification from single cells in RNA-Seq may introduce considerable errors (46). To conclude, we performed transcriptome analysis on three skin-cell types and compared their gene expression patterns. Our analyses may support a series of hypotheses about the genes and pathways that cause diabetic skin lesions. Some genes within these pathways have already been validated as regulators of diabetic dermopathy, whereas other predictions remain to be tested. We anticipate that panels of genes derived from studies of this type will serve as useful biomarkers for clinical diagnosis.

## Conclusion

Normal- and high glucose levels induced cell type-specific shifts in gene expression in three primary skin-cell types, *i.e*., epidermal keratinocytes, dermal fibroblasts, and dermal microvascular endothelial cells. This study uncovers a molecular mechanism of diabetes-related dermatological problems.

## Data Availabilty Statement

The datasets presented in this study can be found in online repositories. The names of the repository/repositories and accession number(s) can be found in the article/**Supplementary Material**.

## Ethics Statement

The study was reviewed and approved by the Animal Ethics Committee of Huazhong University of Science and Technology.

## Author Contributions

YL and QL conceptualized the study. SZ, ZK, and QL developed the methodology. SZ, ZK, CY, PZ, and HJ were in charge of the investigation. SZ, ZK, PZ, LC, and QL wrote the manuscript. QL acquired the funding. CY, YL, and QL provided the resources. CY, YL, and QL supervised the study. All authors contributed to the article and approved the submitted version.

## Funding

This work was supported financially by grants from the National Natural Science Foundation of China (No.81900432), Natural Science Foundation of Hubei Province (2019CFB499), and Science Foundation of Union Hospital (02032018-244).

## Conflict of Interest

The authors declare that the research was conducted in the absence of any commercial or financial relationships that could be construed as a potential conflict of interest.
