# Supplementary material for: High Glucose Causes Distinct Expression Patterns of Primary Human Skin Cells by RNA Sequencing
Source: Front Endocrinol (Lausanne). 2021 Mar 8;12:603645. doi: 10.3389/fendo.2021.603645 (PMC7982678; doi:10.3389/fendo.2021.603645)
Supplement: Supplementary file 1 [file DataSheet_1.pdf]

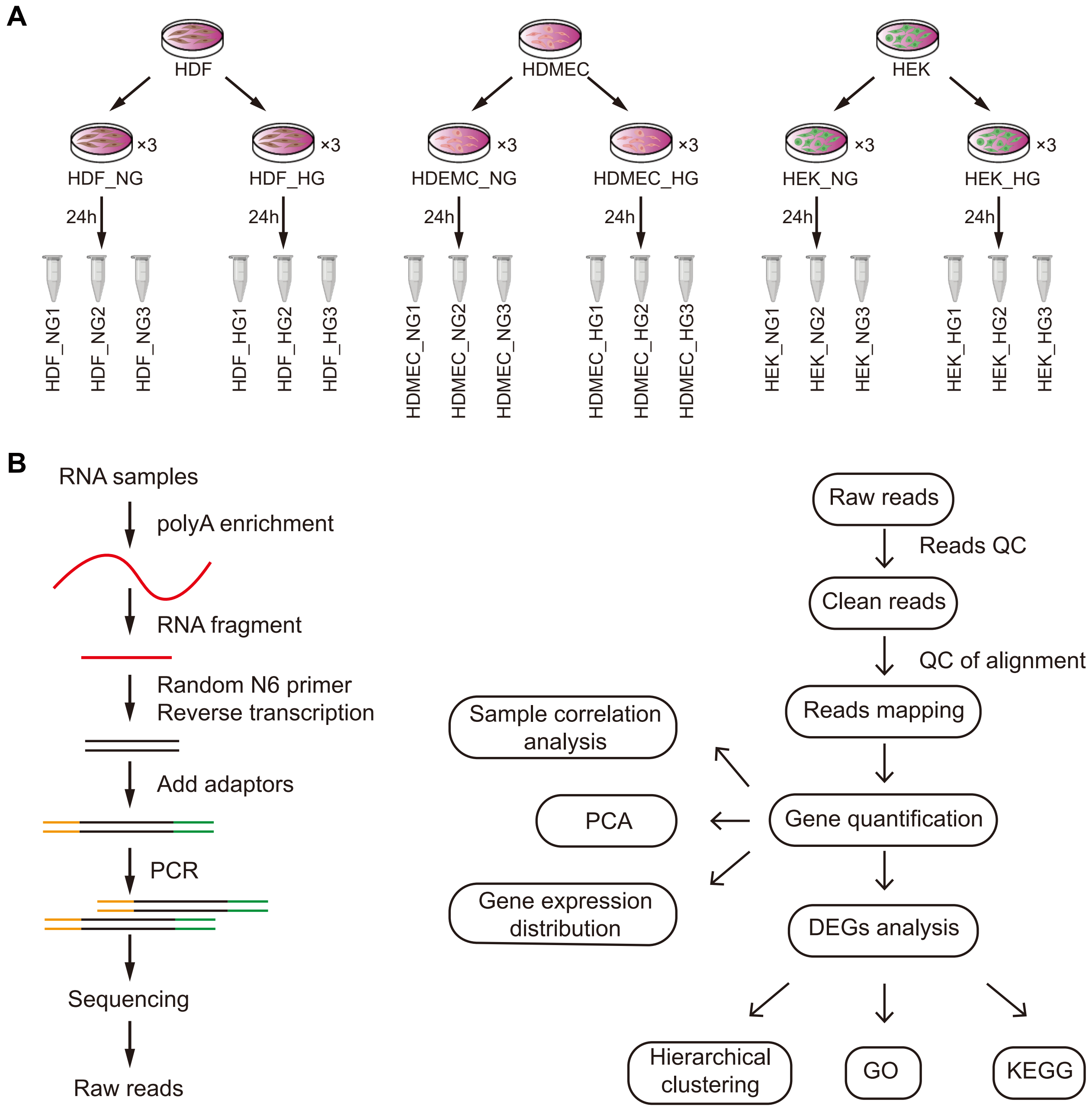

Supplemental Figure 1. Experimental design of transcriptome sequencing.
(A) Human dermal fibroblasts (HDF), human dermal microvascular endothelial cells (HDMEC), and human epidermal keratinocytes (HEK) were treated with high- glucose (HG, 30 mM) or normal glucose (NG) for 24 h, after which total RNA was extracted and subjected to whole-transcriptome sequencing, which was performed in triplicate for each condition.(B) RNA-Seq laboratory workflow and data-analysis pipeline. Two major steps are depicted: (1) Library generation and RNA sequencing of the library; (2) Bioinformatic analysis workflow.

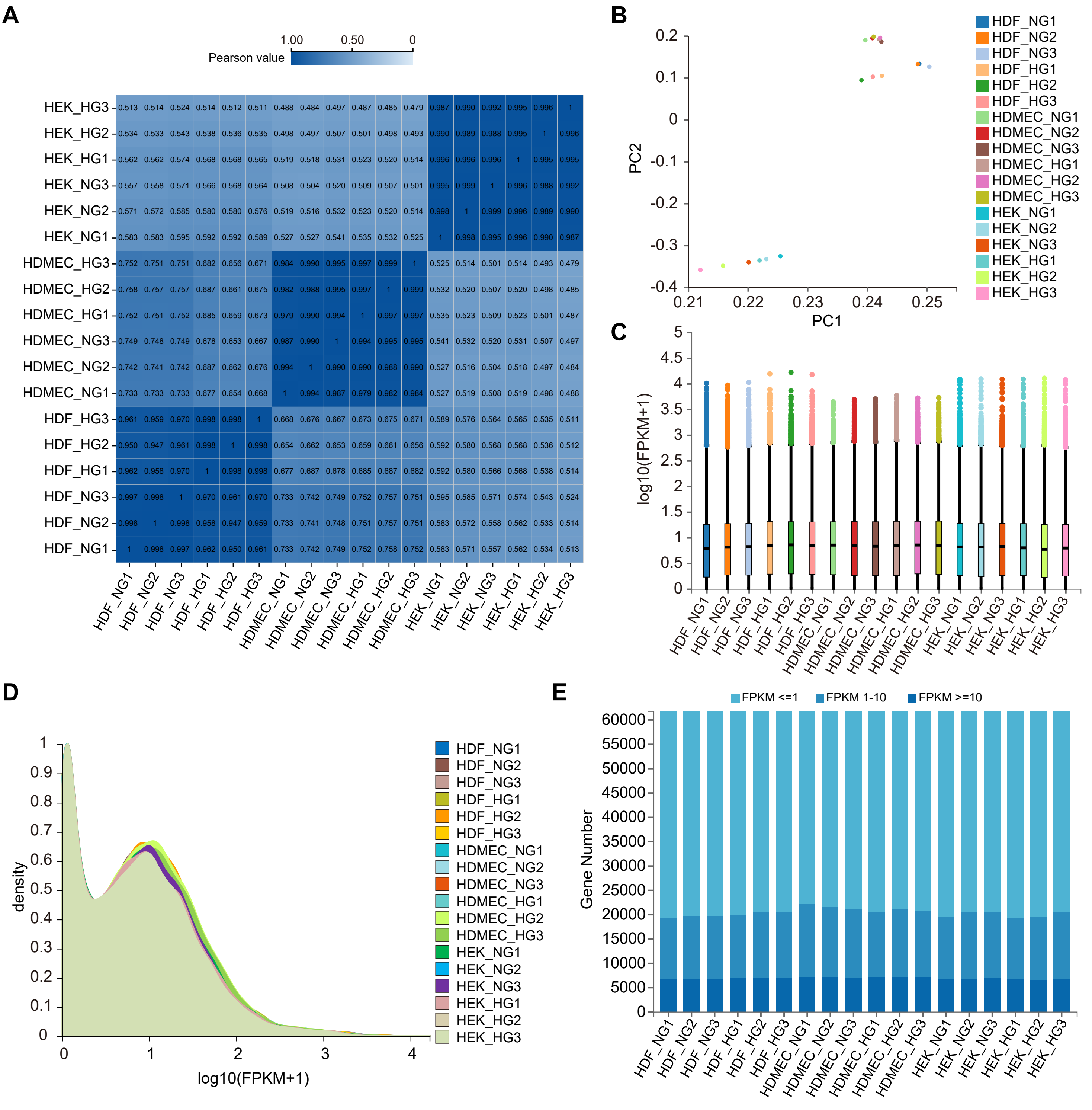

Supplemental Figure 2. Comprehensive analysis of sequencing date

(A) The correlation matrix depicts the correlation of expression profiles for all included genes compared in the three cell types in response to NG or HG stimulation. Heat map colors indicate the height of the Pearson's correlations, which ranged from 0.48 (bluish color code) to 0.99 (blue color code). (B) Principal Component Analysis (PCA) of the 18 sequencing datasets revealed close clustering of biological replicates and separation of the profiles of different cell types. (C) Boxplots showing the distribution of gene expression levels in each sample and the degree of dispersion of the data distribution. (D) Density map showing the interval of gene expression concentration in the samples. (E) Stacked histogram showing the gene number in different FPKM ranges for each sample (FPKM ≤ 1, FPKM 1 ~ 10, FPKM ≥ 10).

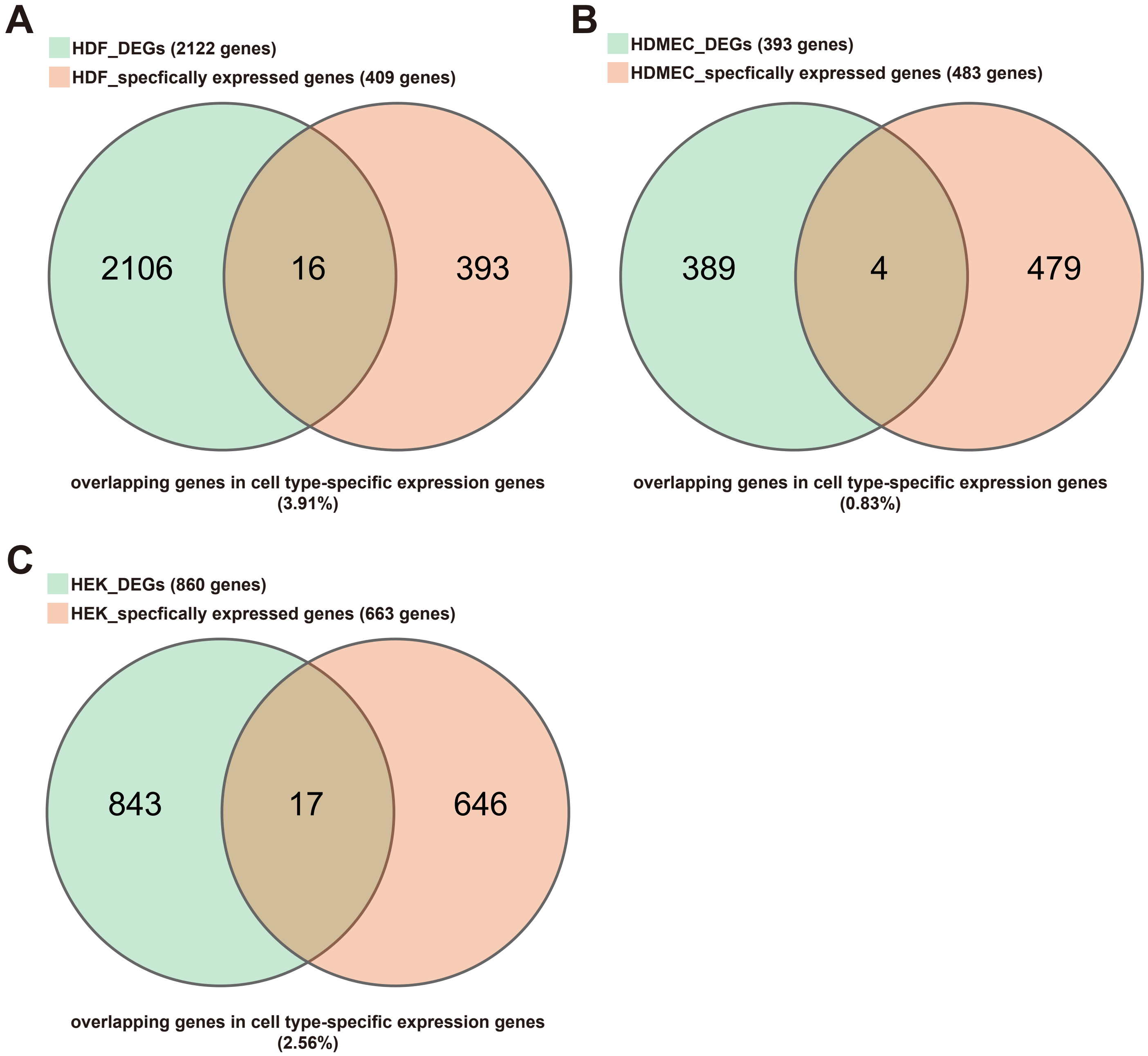

Supplemental Figure 3. Distribution of cell type-specific gene expression changes in each skin-cell type following HG stimulation.

(A-C) Venn diagrams visualizing the overlap between DEGs and cell type-specific expression genes in HDF (A), HDMEC (B), and HEK (C). The ratio is determined by dividing the number of overlapping genes by cell type-specific expression genes.

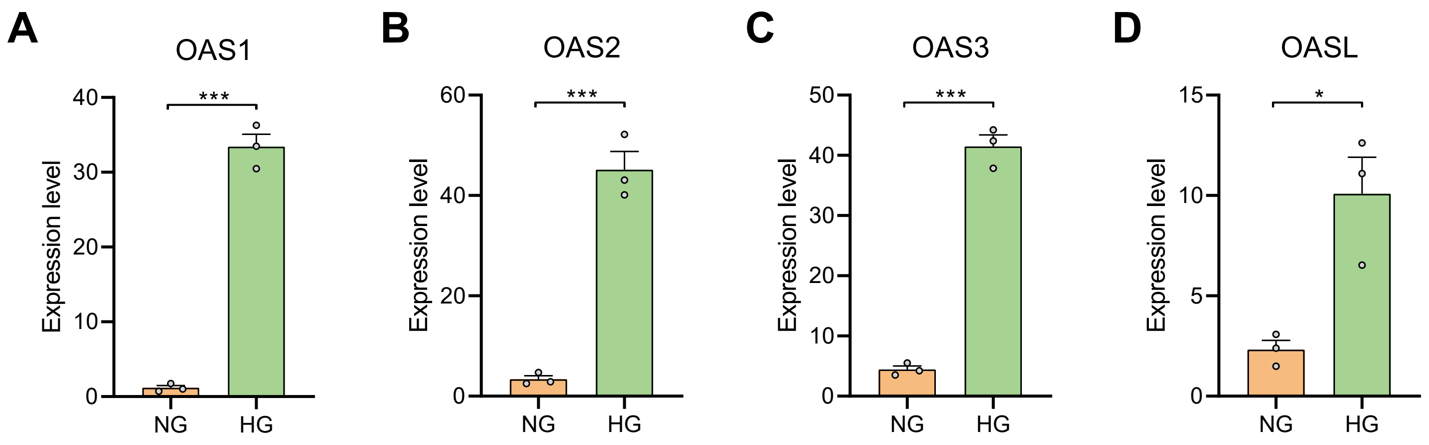

Supplemental Figure 4. Expression levels of OAS family proteins in HG-induced HDF.
(A-D) Histograms depicting gene expression levels of OAS1(A), OAS2 (B), OAS3 (C), and OASL (D) using RNA-sequencing data.

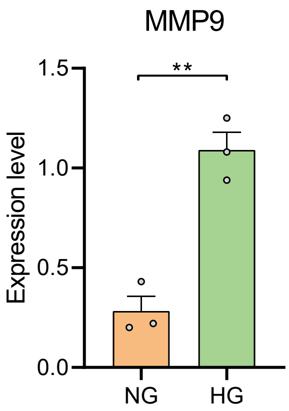

Supplemental Figure 5. Expression levels of MMP9 in HG-induced HDF.
Histograms displaying gene expression levels of MMP9 using RNA-sequencing data.

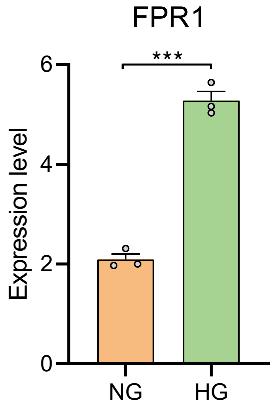

Supplemental Figure 6. Expression levels of FPR1 in HG-induced HEK.
Histograms showing gene expression levels of FPR1 using RNA-sequencing data.

Supplemental Table 1. Detailed information of RNA quality and RNA sample assessment.

Sample	Concentration (ng/μL)	Volume (μL)	Total quantity (μg)	RIN/RQN	28S/18S
**HDF_NG1**	263.2	20	5.26	10.0	1.8
**HDF_NG2**	271.1	30	8.13	10.0	2.2
**HDF_NG3**	273.0	30	8.19	10.0	1.9
**HDF_HG1**	340.1	20	6.8	9.9	1.9
**HDF_HG2**	427.2	30	12.82	10.0	2.2
**HDF_HG3**	369.6	30	11.09	10.0	2.1
**HDMEC_NG1**	100.7	20	2.01	10.0	2.5
**HDMEC_NG2**	123.1	20	2.46	9.7	2.0
**HDMEC_NG3**	115.3	20	2.31	9.0	1.5
**HDMEC_HG1**	113.3	20	2.27	9.0	1.5
**HDMEC_HG2**	154.1	20	3.08	9.1	1.7
**HDMEC_HG3**	153.9	20	3.08	9.3	1.8
**HEK_NG1**	145.2	20	2.9	8.2	2.7
**HEK_NG2**	164.2	20	3.28	8.5	2.6
**HEK_NG3**	202.5	20	4.05	8.4	2.4
**HEK_HG1**	286.5	20	5.73	7.8	2.5
**HEK_HG2**	193.5	20	3.87	8.0	2.2
**HEK_HG3**	199.4	20	3.99	8.5	2.5

Supplemental Table 2. Clean reads information and mapping results of sequencing data.

Sample	Total Raw Reads (M)	Total Clean Reads (M)	Total Mapping Ratio (%)	Uniquely Mapping Ratio (%)
HDF_NG1	49.08	45.28	94.25	89.04
HDF_NG2	51.78	45.18	94.02	88.93
HDF_NG3	50.97	44.63	94.01	88.88
HDF_HG1	50.19	43.6	93.79	88.62
HDF_HG2	51.69	45.03	93.74	88.54
HDF_HG3	52.59	45.86	93.81	88.54
HDMEC_NG1	49.08	45.58	94.55	88.77
HDMEC_NG2	46.02	42.72	94.68	88.7
HDMEC_NG3	49.08	45.85	94.85	88.98
HDMEC_HG1	50.83	47.49	94.51	88.52
HDMEC_HG2	41.06	36.65	88.81	83.74
HDMEC_HG3	45.59	40.82	89.11	83.98
HEK_NG1	41.9	37.5	88.28	83.28
HEK_NG2	49.08	44.87	90.94	85.62
HEK_NG3	45.23	40.19	88.05	83.13
HEK_HG1	41.07	36.2	87.92	83.01
HEK_HG2	52.59	47.35	90.39	84.95
HEK_HG3	41.87	36.72	87.71	82.81
